# An Improved m6A-ELISA for Quantifying *N^6^*-methyladenosine in Poly(A)-purified mRNAs

**DOI:** 10.21769/BioProtoc.5359

**Published:** 2025-06-20

**Authors:** Wei Yee Chan, Waleed S. Albihlal, Folkert J. Van Werven

**Affiliations:** Cell Fate and Gene Regulation Laboratory, The Francis Crick Institute, London, UK

**Keywords:** ELISA, *N* ^6^-methyladenosine, m6A, RNA, mRNA, *Saccharomyces cerevisiae*, Yeast, Mouse

## Abstract

*N^6^*-methyladenosine (m6A) is an abundant internal mRNA modification with roles in regulating cellular and organismal physiology, including development, differentiation, and disease. The deposition of m6A is highly regulated, with various m6A levels across different environmental conditions, cellular states, and cell types. Available methods for measuring bulk m6A levels are often time-consuming, have low throughput, and/or require specialized instrumentation or data analyses. Here, we present a detailed protocol for measuring bulk m6A levels in purified poly(A) RNA samples with m6A-ELISA using a standard-based approach. Critical steps of the protocol are highlighted and optimized, including poly(A) RNA quality controls and antibody specificity testing. The protocol is fast, scalable, adaptable, and cost-effective. It does not require specialized instrumentation, training, or skills in data analysis. We have successfully tested this protocol on mRNAs isolated from budding yeast and mouse cell lines.

Key features

• *N^6^*-methyladenosine quantification, including mRNA isolation, can be achieved in two days.

• Describes a robust method for poly(A) RNA isolation from total RNA, minimizing non-poly(A) RNA contamination.

• Based on Ensinck et al. [1], optimizing poly(A) RNA selection from yeast and mammalian cells and reducing the amount of mammalian mRNA for the assay.

## Graphical overview


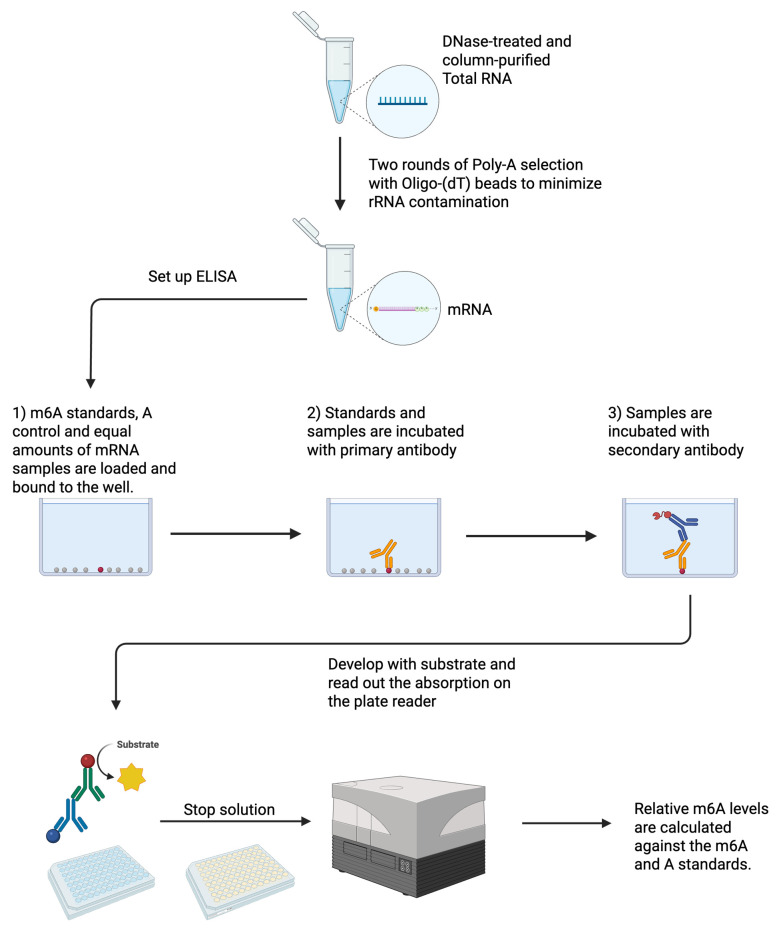


**Total RNA is extracted, DNase-treated, and column-purified.** Two rounds of poly(A) selection are performed to minimize rRNA contamination. Equal amounts of mRNA between samples are bound to wells on a 96-well plate. In vitro transcribed A and m6A are also bound as standards. Samples are incubated with the primary antibody and, subsequently, with the secondary antibody. Samples are developed with a substrate, stop solution is added, and absorption is read out on a plate reader. Relative m6A levels are calculated against the m6A and A standards. *Created in BioRender. Chan, W. (2025)* *https://BioRender.com/e69h636*

## Background

Epitranscriptomics, post-transcriptional chemical RNA base modifications, have emerged as key regulators of gene expression. Among RNA modifications, *N^6^*-methyladenosine (m6A) is a prevalent internal mRNA modification that is deposited by a multisubunit methyltransferase complex (m6A writers) on the RRACH motif sequence in mRNAs [2–4]. The m6A mark is then recognized and bound by various proteins, including a class of proteins containing the YTH (YT521-B homology) domain, also known as m6A readers, and directs m6A-modified transcripts for subsequent fates such as decay, translation, and localization [5–7]. Another group of mRNA-modifying enzymes known as m6A erasers can remove the m6A modification [8]. Together, they make up the m6A machinery, which dynamically regulates m6A levels. As a result, m6A levels may vary substantially across different environmental conditions, cell types, and species. For example, in *Saccharomyces cerevisiae*, m6A is specifically deposited during early meiosis but is absent in other cellular states. The dynamic regulation of m6A is critical for maintaining stem cells and regulating neuronal cell differentiation, development, stress response, and the immune system [9,10]. Misregulation of m6A is implicated in pathology, including that of inflammatory and immune deregulatory diseases and cancer [11].

Various methods for quantifying m6A levels are available, ranging from total or global m6A levels to single-nucleotide m6A quantification. Several sequencing-based approaches exist, including antibody-based approaches (such as m6A-seq [12] and miCLIP [13]), and antibody-free methods (such as GLORI-seq [14], MATZER-seq [15], eTAM-seq [16], and Nanopore [17]). Sequencing methods can be time-consuming, costly, and require specialized data analysis. Global m6A detection methods, on the other hand, do not require sequencing or extensive data analysis, with the most accurate being mass spectrometry (MS), where m6A nucleosides from digested purified mRNA are quantified by LC/MS [18,19]. Thin-layer chromatography [20] and m6A dot blot [21] have also been used for total m6A detection and relative quantification. However, drawbacks of the aforementioned methods include MS requiring specialist training and equipment, whereas thin-layer chromatography and m6A dot blot are typically low throughput and low in sensitivity.

We have previously shown that m6A-ELISA is a simple quantitative method for detecting global m6A levels [1]. m6A-ELISA is a cost-effective and fast approach that does not require specialist training or instruments [1]. Here, we describe a step-by-step protocol of m6A-ELISA, including a detailed description of the procedures for poly(A) selection that yields high-quality mRNA and m6A antibody validation. We optimized the poly(A) mRNA purification step and enhanced the sensitivity for m6A detection.

## Materials and reagents

**Biological materials**

1. DNase-treated and column-purified total RNA from samples to be measured

2. DNase-treated and column-purified total RNA from *ime4*Δ yeast strain (Horizon, catalog number: YSC62750201935429), for antibody blocking. If working with a strain other than BY4743, we recommend generating an ime4 *IME4* deletion strain following the PCR-mediated gene disruption protocol described in [22], *Gene* 1995; Jan 151(1):113–117. https://doi.org/10.1016/0378-1119(95)00144-U. For culture conditions, please refer to [3] *eLife* 2023; NaN:RP87860. https://doi.org/10.7554/eLife.87860

**Reagents**

1. Nuclease-free (NF) water (Ambion, catalog number: AM9932)

2. Lithium chloride, 8 M solution (Sigma-Aldrich, catalog number: L7026)

3. Lithium dodecyl sulfate (LiDS) (Thermo Fisher Scientific, catalog number: 413301000)

4. Tris-HCl, pH 7.4, 1 M (Sigma-Aldrich, catalog number: T21941)

5. EDTA, 0.5 M solution, pH 8.0 (Invitrogen, catalog number: 2624961)

6. 10× PBS (Thermo Fisher Scientific, catalog number: AM9624)

7. Polysorbate 20, e.g., Tween^®^ 20 (Sigma-Aldrich, catalog number: P9416)

8. Oligo-d(T)_25_ magnetic beads (New England BioLabs, catalog number: S1419S)

9. Qubit^®^ RNA HS Assay kit (Thermo Fisher Scientific, catalog number: Q32855)

10. DNA binding microplate solution (Abcam, catalog number: ab156917)

11. *N* ^6^-methyladenosine (m6A) rabbit mAb (ABclonal, catalog number: A19841)

12. Goat anti-rabbit IgG H&L (HRP) (Abcam, catalog number: ab205718)

13. TMB ELISA substrate (fast kinetic rate) (Abcam, catalog number: ab171524)

14. 450 nm Stop solution for TMB substrate (Abcam, catalog number: ab171529)

15. MEGAscript T7 Transcription kit, 25 reactions (ThermoFisher Scientific, catalog number: AM1333)

16. *N^6^*-Methyl-ATP (Jena Bioscience, catalog number: NU-1101L)

17. TURBO^TM^ DNase (ThermoFisher Scientific, catalog number: AM2238)

18. Macherey-Nagel^TM^ NucleoSpin^TM^ RNA Clean-up, Mini kit for RNA clean up and concentration (Macherey-Nagel, catalog number: 740948.250)

**Solutions**

1. Binding buffer (see Recipes)

2. Wash buffer A (see Recipes)

3. Wash buffer B (see Recipes)

4. Primary antibody solution blocked with total m6A-free RNA (DNase-treated and column-purified) *ime4*Δ (see Recipes)

5. Secondary antibody solution (see Recipes)

**Recipes**

*Note: The total volume needed will depend on the number of samples. We recommend all buffers be made up fresh for short-term use and filter-sterilized. The tables below describe the recipes for 50 mL; volumes can be scaled up or down as needed.*

**1. Binding buffer (50 mL)**

**Table:** 

Reagent	Final concentration	Volume
Tris-HCl, pH 7.4, 1 M	20 mM	1 mL
Lithium chloride, 8 M	500 mM	3.125 mL
Lithium dodecyl sulfate (10% w/v)	0.5%	2.5 mL
EDTA, 0.5 M	2 mM	200 μL
NF water		43.175 mL

**Storage:** Short-term (i.e., up to 1 week) at 4 °C.

**Caution:** The LiDS may precipitate at 4 °C; this can be resolved by bringing the solution to room temperature.

**2. Wash buffer A (50 mL)**

**Table:** 

Reagent	Final concentration	Volume
Tris-HCl, pH 7.4, 1 M	20 mM	1 mL
Lithium chloride, 8 M	500 mM	3.125 mL
Lithium dodecyl sulfate (10% w/v)	0.2%	1 mL
EDTA, 0.5 M	2 mM	200 μL
NF water		44.675 mL

**Storage:** Short-term (i.e., up to 1 week) at 4 °C.

**Caution**: The LiDS may precipitate at 4 °C; this can be resolved by bringing the solution to room temperature.

**3. Wash buffer B (50 mL)**

**Table:** 

Reagent	Final concentration	Volume
Tris-HCl, pH 7.4, 1 M	20 mM	1 mL
Lithium chloride, 8 M	500 mM	3.125 mL
EDTA, 0.5 M	2 mM	200 μL
NF water		45.675 mL

**Storage:** Short-term (i.e., up to 1 week) at 4 °C.

**4. Primary antibody solution blocked with total m6A-free RNA (DNase-treated and column-purified) *ime4*Δ (10 mL)**

**Table:** 

Reagent	Final concentration	Quantity
1× PBS 0.1% Tween 20	1×	10 mL
*N* ^6^-methyladenosine (m6A) rabbit mAb	1:10,000	1 μL
Total m6A-free RNA (DNase-treated and column clean-up from *ime4*Δ yeast strain)	500 ng·mL^-1^	5 μg

**Storage:** Short-term (i.e., during setup of the ELISA) at 4 °C. We do not recommend reusing the primary antibody.

**5. Secondary antibody solution (10 mL)**

**Table:** 

Reagent	Final concentration	Volume
1× PBS 0.1% Tween 20	1×	10 mL
Goat anti-rabbit IgG H&L (HRP)	1:5,000	2 μL

**Storage:** Short-term (i.e., during setup of the ELISA) at 4 °C. We do not recommend reusing the secondary antibody.

**Laboratory supplies**

1. Low-retention 300 μL pipette tips (Elkay, catalog number: AER-4REF-S96)

2. Low-retention 1,000 μL pipette tips (Elkay, catalog number: AER-STE1-A18)

3. 10 μL filter pipette tips (Starlab, catalog number: S1121-3810-C)

4. 200 μL filter pipette tips (Starlab, catalog number: S1123-8810-C)

5. 1,000 μL filter pipette tips (Starlab, catalog number: S1126-7710-C)

6. 1.5 mL DNA LoBind Eppendorf tubes (snap cap) (Eppendorf, catalog number: 0030108051)

7. Qubit^®^ assay tubes (Thermo Fisher Scientific, catalog number: Q32856)

8. 96-well microplate pack (Abcam, catalog number: ab210903)

9. Milipore^®^ Stericup^®^ quick release vacuum filtration system (Merck, catalog number S2GPU0SRE)

10. Adhesive PCR plate seals (Thermo Fisher Scientific, catalog number: AB0558)

## Equipment

1. Eppendorf Thermomixer, 24 × 1.5 mL microcentrifuge tubes (or equivalent alternative)

2. DynaMag-2 magnetic rack (Thermo Fisher Scientific, catalog number: 12321D) (or equivalent alternative)

3. Nanodrop One spectrophotometer (Thermo Fisher Scientific, catalog number: ND-ONE-W) (or equivalent alternative)

4. Rotator wheel

5. Multi-channel pipette 30–300 μL (Eppendorf, catalog number: 3125000052) (or equivalent alternative)

6. Qubit 4 fluorometer (Thermo Fisher Scientific, catalog number: Q33238)

7. 450 nm plate reader, e.g., Tecan Spark^®^ microplate reader (or equivalent alternative)

## Software and datasets

1. 450 nm plate reader software, e.g., Tecan Spark Control version 3.0 (or equivalent alternative)

2. Excel Spreadsheet (Microsoft)

3. GraphPad Prism (GraphPad Software) (optional)

## Procedure

**A. Poly(A) RNA pulldown with Oligo(dT)_25_-coupled beads**

1. Introduction:

a. For the following steps, use the DNA low retention tubes (Eppendorf DNA LoBind 1.5 mL microcentrifuge tubes).

b. The low-retention pipette tips should be used when pipetting the beads directly.

c. To reduce rRNA contamination to < 1%, at least two rounds of pulldown should be performed. The first round requires 50 μL of beads per ≥ 50 μg of total RNA, and the second round requires 20 μL.

2. Preparing the beads:

a. Vortex the Oligo (dT) beads thoroughly for a minimum of 5 min. Calculate the total amount of beads required (e.g., 50 μL × 4 samples = 200 μL; add, e.g., 20 μL extra to account for pipetting error). Pipette the beads into a microcentrifuge tube. Vortex the beads between pipetting.

**Caution:** Oligo(dT) beads must be vortexed thoroughly for at least 10 s between pipetting steps to prevent aggregation.

b. Place the beads on the magnetic rack until they are completely separated from the resuspension buffer (approximately 1 min). Then remove the manufacturer’s resuspension buffer. Wash beads at least three times with 1 mL of binding buffer. Then resuspend the beads in binding buffer at the original volume.

**Caution:** It is important to wait for the beads to separate from the buffer on the magnetic rack to avoid bead loss during washing; this can take up to 1 min.

3. Prepare the sample tube:

a. The starting material differs from yeast to mammalian cells. For yeast, a minimum of 50 μg (but ideally, 80 μg) of starting total RNA material. 80 μg of total RNA will yield ~300 ng of high-quality poly(A) RNA, enough for six technical replicates. For mammalian cells, we recommend a minimum of 10 μg. This is enough to yield ~60 ng of high-quality poly(A) RNA, sufficient for six technical replicates. The volume of total RNA should not exceed 100 μL.

b. Add 900 μL of binding buffer to each total RNA sample. Pipette 50 μL of beads into the mix of RNA and binding buffer. Vortex the sample/beads mix vigorously.

4. Place tubes on a rotator wheel, rotate the samples for 15 min at room temperature.

5. Chill samples on ice for at least 2 min.

6. Place tubes onto the magnetic rack. Allow time for the beads to separate from the binding buffer until the buffer is clear; otherwise, leave the samples on the magnet for longer. Remove the binding buffer by pipetting.

7. Wash the beads:

a. Add 1 mL of wash buffer A. Vortex the tube for 10 s minimum. Place it back onto the magnetic rack. Pipette off wash buffer A. Repeat the wash with wash buffer A.

b. Add 1 mL of wash buffer B. Vortex the tube vigorously for at least 10 s. Place it back onto the magnetic rack. Remove wash buffer B. Repeat the wash with wash buffer B.

c. Add 1 mL of 10 mM Tris-HCl. Vortex the tube. Place it back onto the magnetic rack. Remove 10 mM Tris-HCl. Ensure that all the buffers have been removed by doing a short spin in a microcentrifuge and placing them back on the magnetic rack. Remove the small volume of the remaining liquid.

8. Resuspend beads in 55 μL of 10 mM Tris-HCl. After the second round, resuspend the 20 μL beads in 25 μL of 10 mM Tris-HCl.

9. To elute the poly(A) selected RNA, after both pulldown rounds, heat the beads in solution to 75–80 °C, mixing at 1,400 rpm for 5 min in a thermomixer.

10. Immediately place the tube on the magnetic rack, quickly pipette the eluate, and place it into a new 1.5 mL microcentrifuge tube.

**Critical:** Eluates should be transferred immediately to new tubes, and samples should not be allowed to cool down, as this may result in the reassociation of mRNA to Oligo(dT) beads.

11. Quantify eluates:

a. It is good practice to quantify the poly(A)-selected RNA on a Nanodrop after round 1 of poly(A) pulldown before moving forward to round 2.

b. After round 1, from yeast RNA, we would expect in 55 μL of 12–20 ng/μL from 50 μg, 20–35 ng/μL from 80 μg. From mammalian RNA, we would expect 4–6 ng/μL in 55 μL from 10 μg.

c. After round 2, from yeast RNA, we would expect in 25 μL of 12–20 ng/μL from 50 μg, 20–35 ng/μL from 80 μg. From mammalian RNA, we would expect 2–4 ng/μL in 25 μL from 10 μg.

**B. Making A and m6A standards**

**In vitro transcription of unmodified (A) and *N^6^*-methyladenosine (m6A) RNA standards**

1. Follow the manufacturer’s protocol from the MEGAscript^®^ kit.

2. For *N^6^*-methyladenosine (m6A) RNA, replace ATP with *N^6^*-ATP. The ATP solution in the kit is 75 mM per 1 μL, while the *N^6^*-ATP is 100 mM per 1 μL. Instead of 2 μL of ATP in the reaction, use 1.5 μL of *N^6^*-ATP.

The following amounts are for a single 20 μL reaction. Reactions may be scaled up or down if desired (Table 1):

**Table 1.** Reaction volumes for in vitro transcription of unmodified (A) and *N6*-methyladenosine (m6A) RNA standards

Component	Quantity
Nuclease-free water	to 20 μL
ATP solution OR *N^6^*-ATP solution	2 μL ATP solution OR 1.5 μL *N^6^*-ATP
CTP solution	2 μL
GTP solution	2 μL
UTP solution	2 μL
10× reaction buffer	2 μL
Linear template DNA	0.1–1 μg
Enzyme mix	2 μL

3. At the end of the reaction, treat the in vitro–transcribed RNA with 1 μL of TURBO^TM^ DNAse to remove the template DNA. Incubate at 37 °C for 20 min.

4. Quantify the in vitro–transcribed RNA with NanoDrop. The m6A-modified RNA should be quantified with Qubit only, since NanoDrop does not quantify m6A-modified RNA accurately.

5. Perform the appropriate RNA column clean-up.

6. After clean-up and elution, aliquot the RNA into appropriate-sized aliquots to complete the standards for one plate. We recommend a minimum of 1,200 ng per aliquot for in vitro adenosine to allow for three replicates of each standard. For in vitro m6A, we recommend a concentration of 10 ng/μL in a 10 μL aliquot.

7. Store at -80 °C.

**Performing the ELISA**

1. We use a 96-well microplate. Add 90 μL of DNA binding solution to each well that will be used for standards and samples. We recommend three technical replicates, but the minimum is two.

2. Pipette 1 μL of serially diluted in vitro–transcribed m6A-RNA (see Table 2) into the ELISA plate. The A is required to block the background signal of the primary m6A antibody; therefore, it is added to every m6A-RNA standard. The standards allow for the calculation of a linear equation and are a positive control for the primary antibody.

**Table 2.** Amounts of A and m6A to form the m6A-RNA standard

Standard values
50 ng A + 0.02 ng m6A
50 ng A + 0.01 ng m6A
50 ng A + 0.0075 ng m6A
50 ng A + 0.005 ng m6A
50 ng A + 0.0025 ng m6A
50 ng A + 0.00125 ng m6A
50 ng A + 0.0006 ng m6A
50 ng A

3. The recommended concentrations for standards are shown in Table 2. They can be changed according to the amount of poly(A) tail-selected RNA loaded onto the plate. The 450 nm absorbance of all samples being tested should lie within the dynamic range of the standards. All standards should be quantified using the Qubit^®^ RNA HS Assay kit.

4. Sample loading:

a. For yeast samples, add 50 ng of mRNA to each well. For mammalian samples, add 10 ng of mRNA to each well.

**Caution:** There is a volume threshold per well. The volume of mRNA sample of both yeast and mammalian should not exceed 10 μL per well (to make up the 50 or 10 ng, respectively), which will be added to the 90 μL binding solution, for a maximum final volume of 100 μL.

b. Use the multichannel pipette with 200 μL pipette tips. Set it to 50 μL and gently mix the DNA binding solution with the loaded standards and samples thoroughly. Avoid splashes and bubbles. Bubbles can be disrupted using a needle.

**Figure 1.**
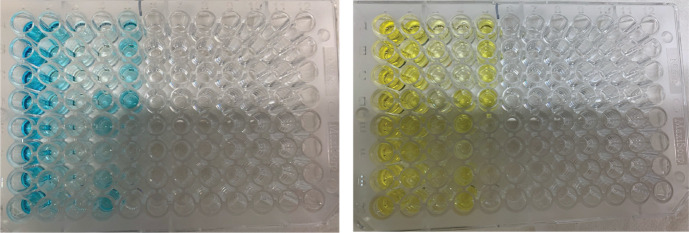
Example of an m6A-ELISA signal in a 96-well microplate. On the left is the blue color from the developing substrate solution. On the right is the color change once the stop solution has been added.

5. Cover the microplate with its manufacturer-provided adhesive film. Ensure the plate is sealed properly to avoid evaporation.

6. Incubate at 37 °C for 2 h.

7. Remove the binding solution (containing standards and samples) with a multichannel pipette.

8. Wash each well with 200 μL of PBS-Tween 0.1% using a multichannel pipette. This involves filling each well with the solution and throwing the solution off by flicking the plate over the sink. Do these three more times. Dry the plate by tapping it lightly on a tissue.

9. Add 100 μL of primary antibody solution to each used well. Remove bubbles by gentle tapping or by using a needle. Incubate at room temperature for 1 h.

10. Wash each well with 200 μL of PBS-Tween 0.1% four times. Dry the plate as before.

11. Add 100 μL of secondary antibody solution to each well. Remove bubbles. Incubate at room temperature for 30 min.

12. Wash each well with 200 μL of PBS-Tween 0.1% five times. Dry the plate as before.

13. Develop:

a. Add 100 μL of fast kinetic ELISA substrate to each well with a multichannel pipette. Do this as swiftly as possible. Incubate at room temperature for 25–30 min. There is no exact time for the development of the substrate, and incubation up to 1 h is possible. Do not overdevelop the standards to saturation. It is possible to judge this by the blue color of the ELISA substrate (see Figure 1). We found that a development time of up to 30 min is adequate for yeast and mammalian RNA samples.

b. After adequate development, add 100 μL of stop solution. This will turn the blue-colored developing solution yellow and stop development (Figure 1).

14. Read out the ELISA on a plate reader at 450 nm.

**Caution:** Ideally, absorbance measurements should be taken within 30 min of stopping the development. The colorimetric reaction is stable after the addition of the stop solution. Evaporation of samples may occur if the plate is left at room temperature for a long time, which could change the readout values. We would recommend covering the plate with an adhesive plate seal or parafilm to avoid evaporation if there is a need to wait longer for a plate reader.

## Data analysis

1. Calculate the mean or median of each technical replicate for each standard and each sample.

2. Correct the values by subtracting the background signal (acquired from the mean or median of the A value).

3. Calculate a linear equation from the standard curve. This can be done in Excel by adding a linear trendline and then adding the equation from the trendline menu.

4. Apply the linear equation to the samples.

5. The bar graph displaying the values can be plotted in Excel or GraphPad Prism. Statistical analyses of biological replicates can be performed on GraphPad Prism.

## Validation of protocol

**Figure 2.**
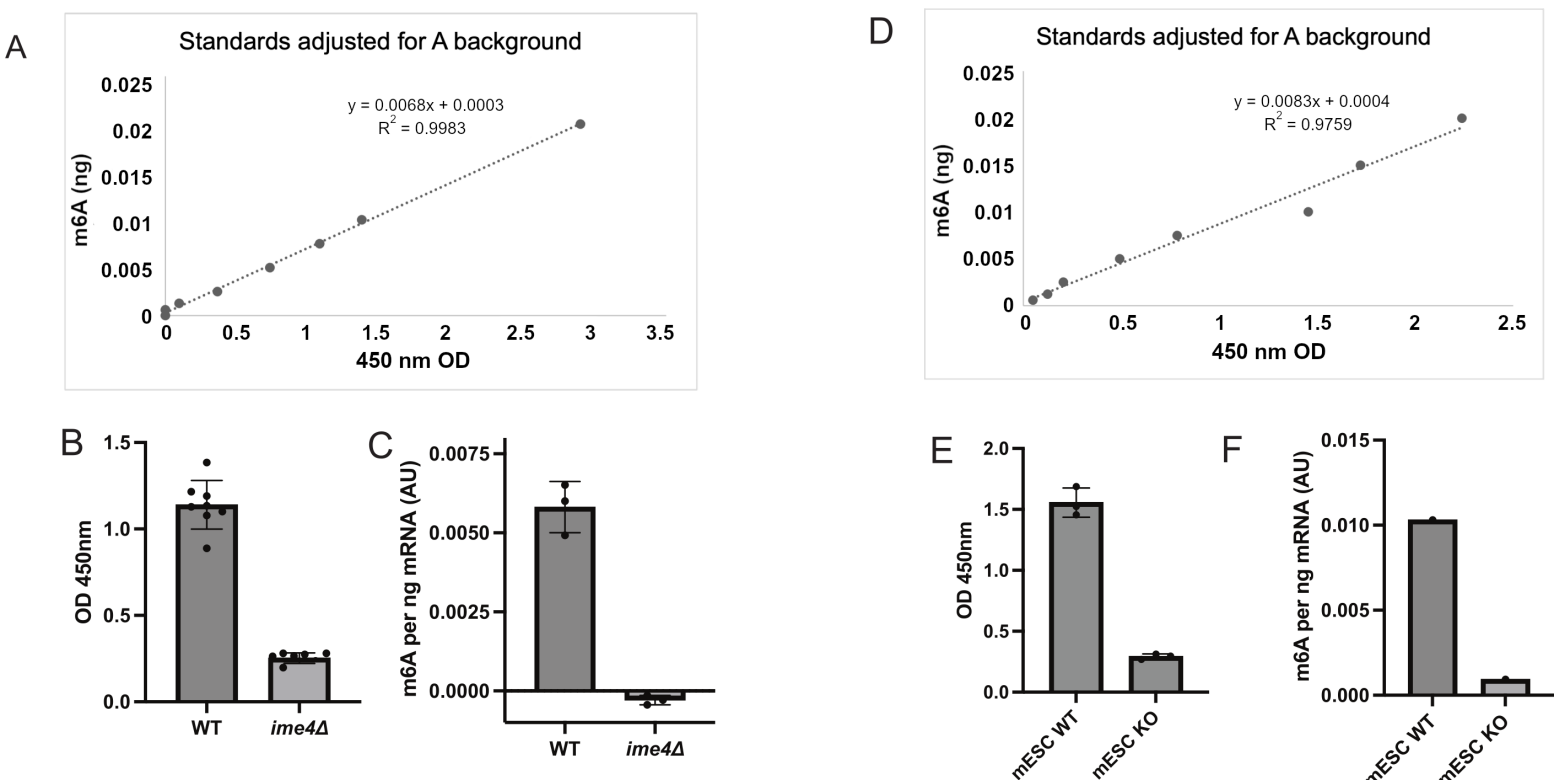
Quantification of m6A using the m6A-ELISA protocol on poly(A) RNA isolated from yeast and mouse embryonic stem cells (mESCs). (A) The m6A standard was generated by an in vitro transcription reaction with m6A or A (background control) DNA template (included in the MEGAscript^®^ kit). The graph shows the m6A standard (ng), on the y-axis, to the absorbance values (OD 450 nm), on the x-axis. (B) Poly(A) RNA isolated from wildtype (WT) and *ime4*Δ cells staged in early meiosis. The technical replicates are plotted for 3 biological replicates. Shown are absorbance values (OD 450 nm) acquired from the plate reader. The mean is plotted. The error bars represent the standard deviation. (C) The same data as in B, where the equation from the m6A standards has been applied to the mean of each biological replicate. Shown is the mean of n = 3 plus standard deviation. (D) The m6A standard curve for the experiment described in E and F. (E) Poly(A) isolated from mESC wildtype or knock out (KO) for METTL3. Shown are 3 technical replicates of OD 450 nm values. (F) The same data as E, except that the equation from the standards (D) was applied to the average of a biological replicate.

1. Sensitivity and specificity of the recommended primary antibody (ABclonal, catalog number: A19841) have been tested and compared between different manufacturers [Ensinck et al. [1]. m6A-ELISA, a simple method for quantifying N6-methyladenosine from mRNA populations. *RNA* (Figure 1D)]. Increasing specificity can be achieved by blocking the antibody with total m6A-free RNA isolated from *ime4*Δ cells. Other m6A-antibodies may work too, depending on their sensitivity and specificity to the m6A modification. We recommend validating new antibodies with a wild-type control and *ime4*Δ equivalent in a yeast sample.

2. The sensitivity and specificity of the m6A-ELISA between wildtype and *ime4*Δ have also been compared to results obtained from LC/MS [Ensinck et al. [1]. m6A-ELISA, a simple method for quantifying N6-methyladenosine from mRNA populations. *RNA* (Figure 2C); Varier et al. [23]. *N* ^6^-methyladenosine (m6A) reader Pho92 is recruited co-transcriptionally and couples' translation to mRNA decay to promote meiotic fitness in yeast. *eLife* (Figure 5G)].

3. The m6A-ELISA has also been used to study the methyltransferase complex in yeast [Ensinck et al. [3]. The yeast RNA methylation complex consists of conserved yet reconfigured components with m6A-dependent and independent roles. *eLife* (Figure 2C)].

4. We have also separately validated the poly(A)-selected RNA from the new and improved Oligo(dT) poly(A) pulldown protocol written here by performing the m6A-ELISA on both yeast samples (WT vs. *ime4*Δ) and mammalian samples [(mouse embryonic stem cells (WT vs. METTL3 KO)] (Figure 2).

## General notes and troubleshooting

**General notes**

1. Total RNA isolated from yeast *ime4*Δ cells (m6A-free RNA) can be used as a blocking reagent for the primary antibody solution when testing mammalian samples.

2. Due to the nature of an antibody-based ELISA, it is not always possible to avoid plate-to-plate variability. This may be attributed to batch-to-batch differences in the reagents used. Therefore, it is best practice to include a WT and *ime4*Δ (or equivalent) controls. Besides acting as reference samples for relative quantification, they can also be used as quality control samples.

3. m6A-ELISA is not for absolute quantification of m6A levels; it is only used for relative bulk m6A quantification between samples.

**Troubleshooting**

**Oligo(dT) beads and poly(A) selection**

1. It is important to thoroughly vortex Oligo(dT) beads before use, as they tend to sediment and aggregate, which can affect the volume of beads and, in turn, affect the efficiency of capture.

2. Oligo(dT) beads take time to accumulate onto the magnet. It is important not to rush the removal of the binding and wash buffers. Allow enough time for the solutions to become completely clear, and ensure that no brown color remains in the solution. It is possible to lose a significant amount of poly(A)-selected RNA from the beads being lost in the wash steps if they have not finished accumulating on the magnet.

3. The ratio of total RNA to beads has been optimized. We would recommend testing any changes to this ratio before use.

4. Swift action at heat elution is important. Allowing the samples to cool off may lead to a significant amount of mRNA rebinding to the beads, which will affect the yield.

**ELISA**

1. Avoid bubbles throughout, as this can affect the technical replicates of the samples.

2. Thoroughly mixing the samples into the binding buffer by gentle pipetting is important to ensure the reproducibility of the plated technical replicates.
